# Evaluating the effect of the incidence angle of ALOS-2 PALSAR-2 on detecting aquaculture facilities for sustainable use of coastal space and resources

**DOI:** 10.7717/peerj.14649

**Published:** 2023-01-06

**Authors:** Hiroki Murata, Toyonobu Fujii, Chinatsu Yonezawa

**Affiliations:** 1 Research Center for Advanced Science and Technology, The University of Tokyo, Tokyo, Japan; 2 Graduate School of Agricultural Science, Tohoku University, Sendai, Japan

**Keywords:** Satellite remote sensing, L-band SAR, Aquaculture facility, Coastal management, Ago Bay, Mie Prefecture, Central Japan

## Abstract

**Background:**

Driven by the growing world population, aquaculture plays a key role in meeting the increasing demand for food. However, aquaculture facilities in Japan are widely installed in coastal waters where natural disasters, such as typhoons and tsunamis, might wash these facilities away, thereby interfering with maritime navigation safety. Therefore, it is imperative to efficiently monitor the state of aquaculture facilities daily, particularly after a disaster in real time. To this end, several new space-borne L-band synthetic aperture radars (SARs) continue to be launched now and in the future, whose utilizations are expected to increase nationally and internationally. An example is the Japan Aerospace Exploration Agency, currently operating a SAR that can be operated day and night, and even under cloudy conditions, called ALOS-2 PALSAR-2.

**Methods:**

Based on the above facts, this study evaluated the effect of the incidence angle of ALOS-2 PALSAR-2 HH single-polarization data, using 3 m spatial resolution, on aquaculture raft detection. As the study site, we selected Ago Bay, located on the Pacific coast of Mie Prefecture in central Japan since the Nankai Trough Megathrust Earthquake and tsunamis have been predicted to occur in the future around this area. Then, we analyzed the sigma zero (backscattering coefficient) of aquaculture rafts and their surrounding sea surfaces, including the relationships between satellite orbits and aquaculture raft directions.

**Results:**

Investigations revealed that the optimum incidence angle for detecting aquaculture rafts in this study was 33.8°–45.1°. Differences in the sigma zero values existed between the ascending and descending orbits. However, the incidence angles differed on the orbits. Then, differences in the median sigma zero values across a range of incidence angles were evaluated under the descending orbit. In addition, when the directions of the aquaculture rafts were closely perpendicular to the satellite orbit, aquaculture rafts tended to show the highest values of sigma zero due to Bragg resonance scattering. Hence, this knowledge may allow for the rapid detection of aquaculture rafts during an emergency without going on-site.

## Introduction

Sustainable food production through aquaculture is expected to correspond to the increasing food demand of the growing world population in coastal spaces (FAO, 2022). In Japan, coastal aquaculture has widely been practiced and recognized as one of the primary industries with its economic ripple effects generating income and employment in, for example, seafood processing, tourism, and transportation sectors. Therefore, aquaculture is key to implementing the sustainable development of coastal spaces. Aquaculture adopt several types of cultivation facilities within sea surface areas, allocated through the fishing right and licensing management systems based on the Fisheries Act (Asada, Hirasawa & Nagasaki, 1983). However, although these fishery cooperative associations effectively manage the spatial plan of aquaculture facilities in each region, their exact locations have not been well documented (Murata et al., 2021), and information about their actual states, including numbers, has not been publicly made available.

Coastal waters play an important role during a disaster. In Japan, for example, national and local governments have established plans to ship emergency relief supplies, such as food and clothing, through these routes after a disaster such as an earthquake and tsunami (*e.g*., Cabinet Office, Government of Japan, 2021; Shima City, Shima City Disaster Prevention Council, 2021). Hence, assessing the situation in coastal waters to determine whether vessels can safely navigate through the port after a disaster is necessary. Furthermore, when a disaster, especially a tsunami occurs, fishing communities are anxious to know about the state of their aquaculture facilities and culturing products. Still, they are often not allowed to go near the sea for a while after a tsunami due to safety reasons. For instance, a tsunami may wash away aquaculture facilities (Suppasri et al., 2018), making vessel navigation difficult. Therefore, it is necessary to find methods of daily and immediately comprehending aquaculture facilities’ status after a disaster without visiting the site, which aids information sharing among stakeholders.

Remote sensing could help solve the aforementioned issue. This technology observes ground surface conditions from satellites, airplanes, drones, *etc*., using sensors. Based on their high spatial resolution, sensors can mainly be divided as optical sensors and synthetic aperture radars (SARs). Although optical sensors receive reflected sunlight, being able to observe during the daytime but not under clouds, SAR irradiates and receives microwaves. Therefore, they can observe ground objects regardless of the time of day. Furthermore, since microwaves can penetrate clouds, observation under clouds is possible with SAR (Ottinger & Kuenzer, 2020), making it a powerful tool for emergency observations immediately after a disaster. Consequently, several studies have employed SAR to detect aquaculture facilities. For example, while Zhang et al. (2022) proposed a method for extracting marine raft aquaculture areas using C-band SAR Sentinel-1 images by analyzing the features of marine surface areas in China, Gao et al. (2022) proposed the D-ResUnet model for extracting the floating raft information of aquaculture areas from Sentinel-1 images in China. Notwithstanding, these studies primarily focused on image analysis methods for detecting aquaculture areas and not on the observation conditions or the state of individual facilities.

An agency in Japan, the Japan Aerospace Exploration Agency (JAXA), collaborated with domestic and overseas institutions to improve on these issues by assessing the impacts of the 2011 Great East Japan Earthquake on local conditions, using earth observation satellites (Kaku, Aso & Takiguchi, 2015). It is considered preferable to use space-borne SAR in its own country as much as possible, because it is preferable for emergency observations of its flexibility. Hence, space-borne L-band SAR ALOS-2 PALSAR-2 has been operated since 2014, with plans to launch a new L-band SAR ALOS-4 PALSAR-3 in the next few years. This proposed technology is also expected to operate continuously when a disaster occurs. Previous studies using ALOS-2 PALSAR-2 in coastal spaces have reported the detection of shorelines (Asaka et al., 2020), flooded areas (Kwak, 2018; Ohki et al., 2020), ships (Park et al., 2018; Sonoke & Demura, 2020), and aquaculture areas (Murata et al., 2019a). Murata et al. (2019a) studied in Hirota Bay, Iwate Prefecture, Japan, applying ALOS-2 PALSAR-2 full polarimetric data which spatial resolution was 6 m. However, they could not identify individual aquaculture facilities due to their physical sizes relative to the satellite’s spatial resolution. The size of aquaculture raft in Hirota Bay was approximately 4 m × 10 m, as measured by Google Earth (Google, Mountain View, CA, USA). In yet another study, Murata, Komatsu & Yonezawa (2019b) applied L-band airborne SAR Pi-SAR-L2 with a pixel resolution of 1.76 m × 3.2 m to successfully detect three aquaculture facilities in Matsushima Bay, Miyagi Prefecture, Japan. The sizes of the individual raft, longline, and rack aquaculture facilities were approximately 5 m × 15 m, 1 m × 60 m, and 2–5 m × 60 m, respectively. Therefore, based on these previous studies, we anticipated an enhanced capability of detecting aquaculture facilities using the ALOS-2 PALSAR-2 spatial resolution 3 m data. Still, there has been no study demonstrating individual aquaculture raft detection using ALOS-2 PALSAR-2 to date. Additionally, there have only been a few reports, except for Singha, Johansson & Doulgeris (2020), investigating incidence angle dependence using ALOS-2 PALSAR-2. Hence, this study evaluated the effect of the incidence angle of ALOS-2 PALSAR-2 on detecting individual aquaculture facilities.

## Materials and Methods

### Study area and aquaculture rafts

**Figure 1:**
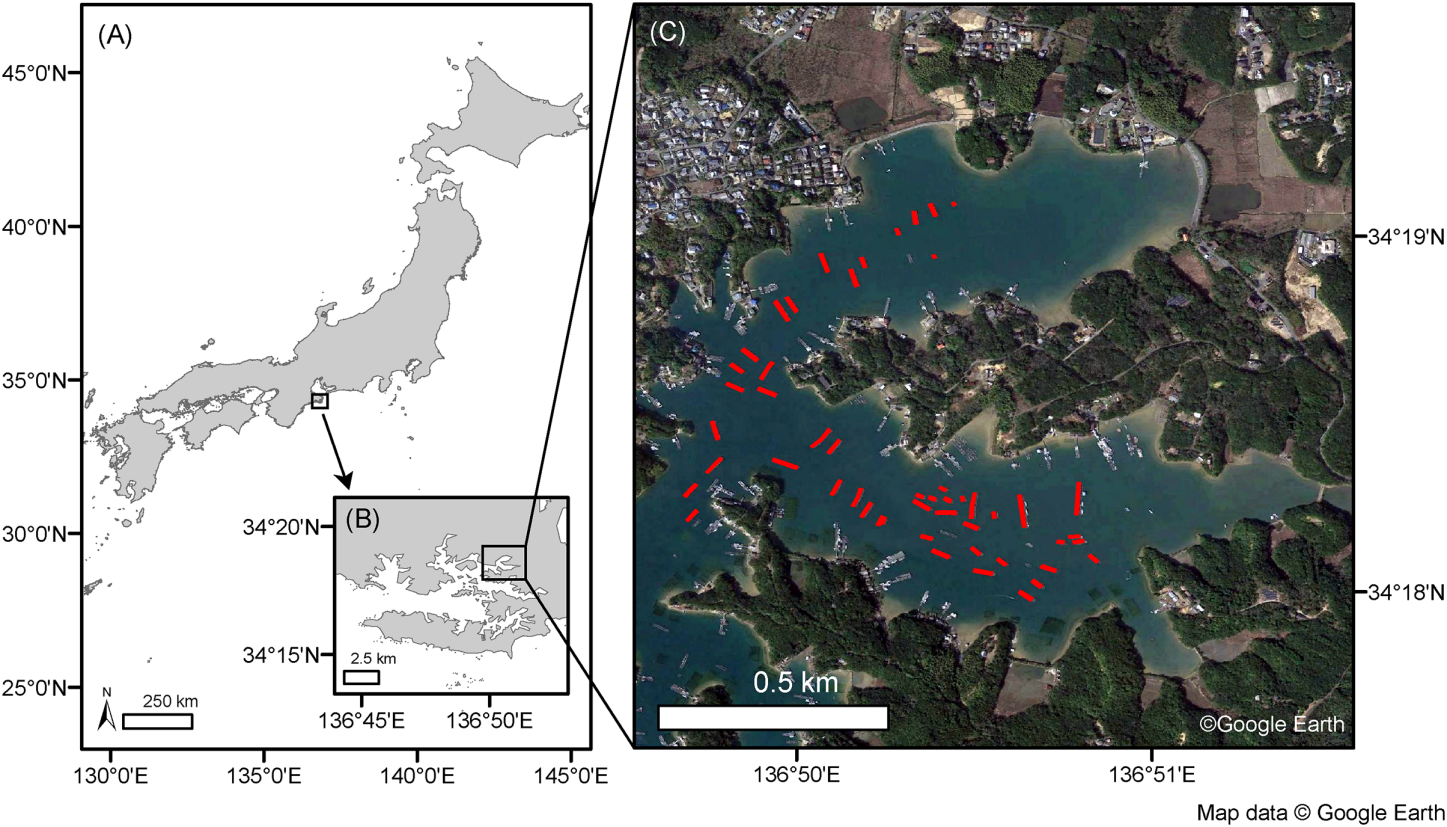
Map of the study site. Map shows the study site’s location in the inner part of Ago Bay, Mie Prefecture, central Japan (A, B). Red polygons show aquaculture rafts mapped from a Google Earth image on March 15, 2021, by visual interpretation (C). Map data (C) copyright belongs to Google Earth.

**Figure 2:**
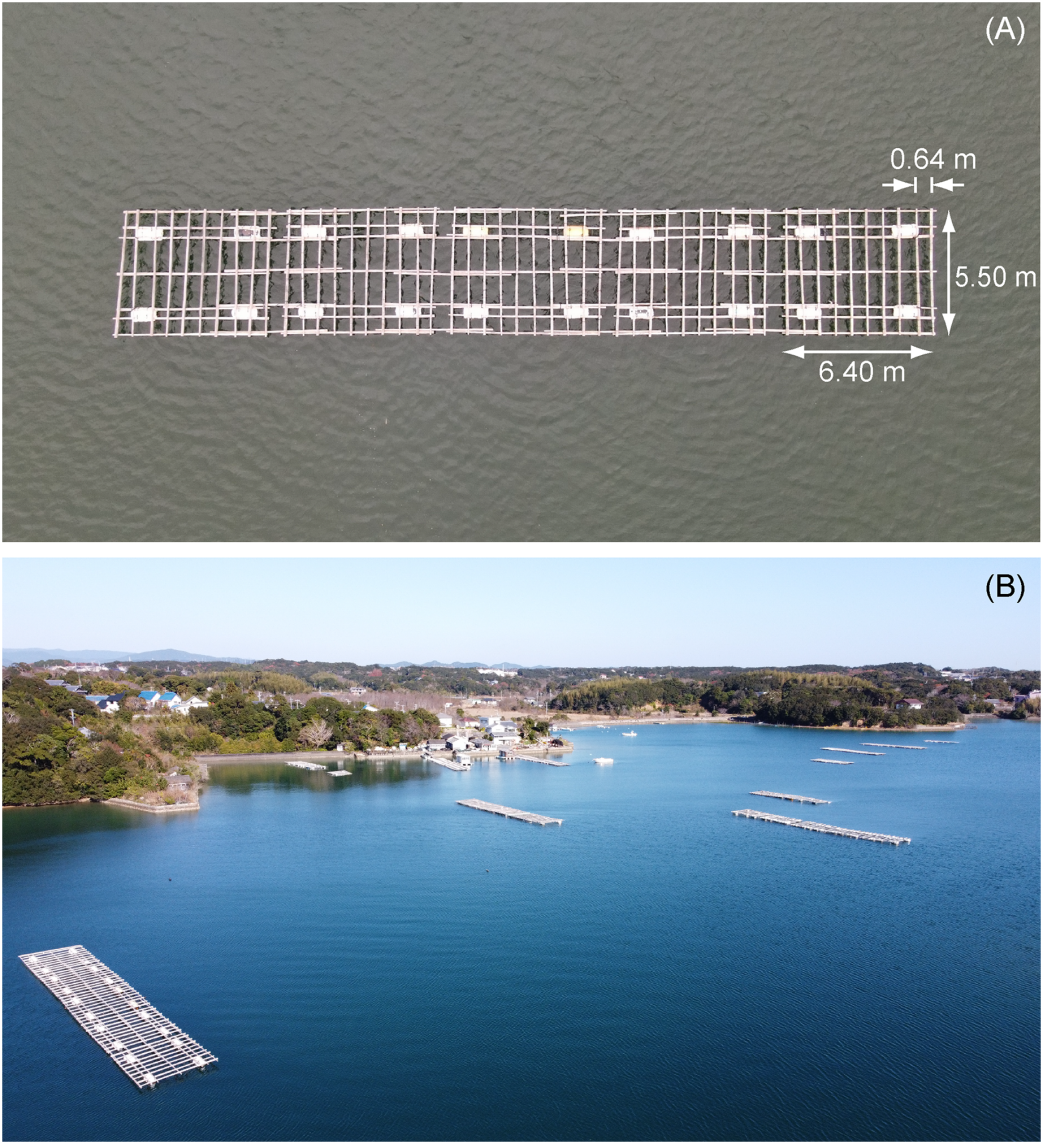
Images of aquaculture rafts. An aquaculture raft taken from a drone Mavic 2 Zoom (DJI, China) on August 29, 2022 (A). Aquaculture rafts in the northern part of the study site, taken from a drone Mavic Mini (DJI, China) on December 16, 2021 (B).

The selected study site was the inner part of Ago Bay, located in the central Japanese prefecture of Mie (Fig. 1). This area has been subject to the Nankai Trough Megathrust Earthquake and tsunamis, with historical accounts of earthquake and tsunami damages recorded along the Nankai Trough in 1498, 1361, 1096, 887, and 684 CE, coupled with the prediction of future occurrences (Fujino et al., 2018). Therefore, recording the aquaculture facilities’ type, number, and location in advance has been considered important. Notably, aquaculture rafts used here were constructed using Hinoki cypress (*Chamaecyparis obtusa*) wood poles and buoys, as shown in the presented example image observed by drone Mavic 2 Zoom (DJI, Nanshan, SZ, China) on August 29, 2022 (Fig. 2A), with results showing that they connected several 5.5 m × 6.4 m rafts, each of which has varying overall raft sizes (Sawada, 1965). These have been used to cultivate the Japanese pearl oyster, *Pinctada fucata* (Gould, 1850), and the oyster, *Magallana gigas* (Thunberg, 1793).

### ALOS-2 PALSAR-2 data and data analysis

**Figure 3:**
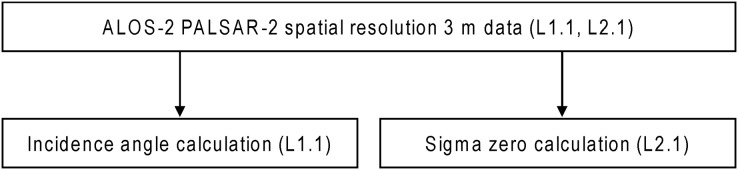
Flowchart of data procedure for ALOS-2 PALSAR-2 data analysis.

We subsequently analyzed ALOS-2 PALSAR-2 data obtained under the ultra-fine beam mode with HH polarization in 2021. The spatial resolution used was 3 m, and the pixel resolution was 2.5 m. To this end, we first studied the data differences between the satellite’s ascending (Asc.) and descending (Desc.) orbits, after which two datasets with the same incidence angles were used to confirm whether the data were peculiar or not at different seasons (Table 1). Next, we examined the differences due to incidence angles, using four datasets with different off-nadir angles under the Desc. orbit (Table 2). Since the incidence angle increased outward from a near to far range in a SAR image, and the difference in the incidence angle between the near and far range sides of the study area in the observation image was less than 0.1, we adopted the center incidence angle for the results and discussion. Then, the incidence angles of each ALOS-2 PALSAR-2 L1.1 data were collected using the ESA SNAP 8.0 software (flowchart is shown in Fig. 3). The results indicated in the applied datasets, as shown in Table 2. Afterward, we calculated the sigma zero ( ${\sigma ^0}$) (also called the backscattering coefficient) for analysis, following conversion with the following equation:

**Table 1:** Dataset used to study differences in ascending (Asc.) and descending (Desc.) orbits. Incidence angles are shown in the near, center, and far ranges.

Polarization	Orbit	Incidence angle (°)			Date
		Near	Center	Far	
HH	Asc.	40.1	40.1	40.1	March 3, 2021
					November 24, 2021
	Desc.	44.0	44.0	44.1	March 23, 2021
					December 28, 2021

**Table 2:** Dataset used to study differences between incidence angles under descending (Desc.) orbit. Incidence angles are shown in the near, center, and far ranges.

Polarization	Orbit	Incidence angle (°)			Date
		Near	Center	Far	
HH	Desc.	33.8	33.8	33.8	August 1, 2021
		44.0	44.0	44.1	September 7, 2021
		45.0	45.1	45.1	May 25, 2021
		52.1	52.1	52.2	December 9, 2021

(1) $${\sigma ^0} = 10\cdot lo{g_{10}}\langle D{N^2}\rangle + CF$$

where DN is the digital number of data and CF is the calibration factor, defined as a constant value of −83 dB (JAXA, 2017).

First, we mapped 50 aquaculture rafts (shown in red polygons in Fig. 1C), after which we randomly selected 50 other areas without aquaculture rafts (sea surface) through visual interpretation based on a Google Earth image collected on March 15, 2021. In second, we analyzed the ALOS-2 PALSAR-2 L2.1 data and collected DN using the ENVI 5.5 (Harris Geospatial, USA) software. The aquaculture rafts direction to satellite orbit direction was also collected using ENVI 5.5 software. Finally, we launched the drone Mavic Mini (DJI, Nanshan, SZ, China) on December 16, 2021, with results confirming that the 50 aquaculture rafts were still in place (a part shown in Fig. 2B).

## Results

### Differences in sigma zero between the Asc. and Desc. orbits

**Figure 4:**
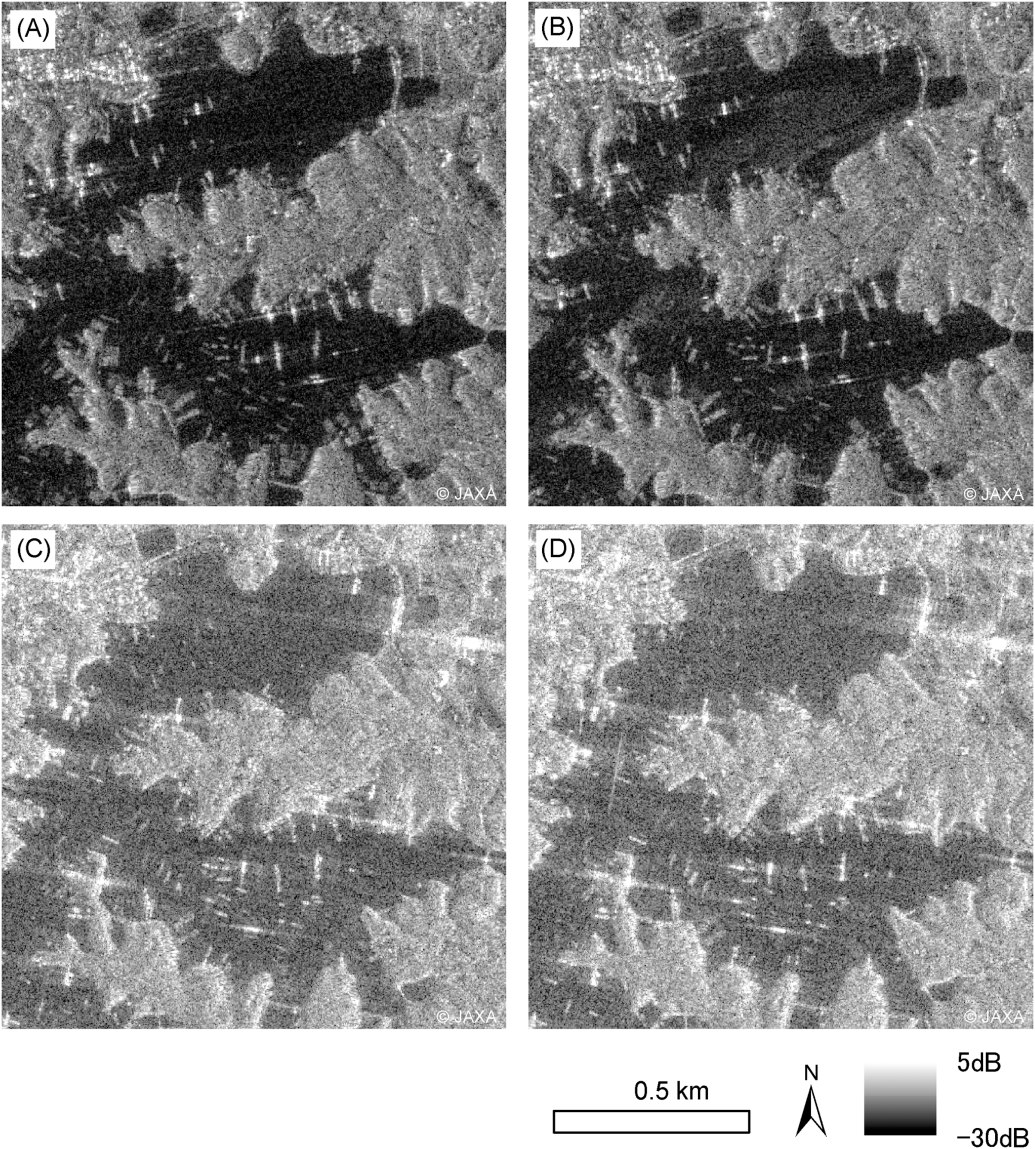
ALOS-2 PALSAR-2 HH single polarization images. Images on March 3, 2021 (A), and November 24, 2021 (B), in which center incidence angles were 40.1° in ascending orbit observation. ALOS-2 PALSAR-2 HH single polarization images on March 23, 2021 (C), and December 28, 2021 (D), which center incidence angle 44.0° in descending orbit observation. ALOS-2 PALSAR-2 data copyrights belong to Japan Aerospace Exploration Agency (JAXA).

**Figure 5:**
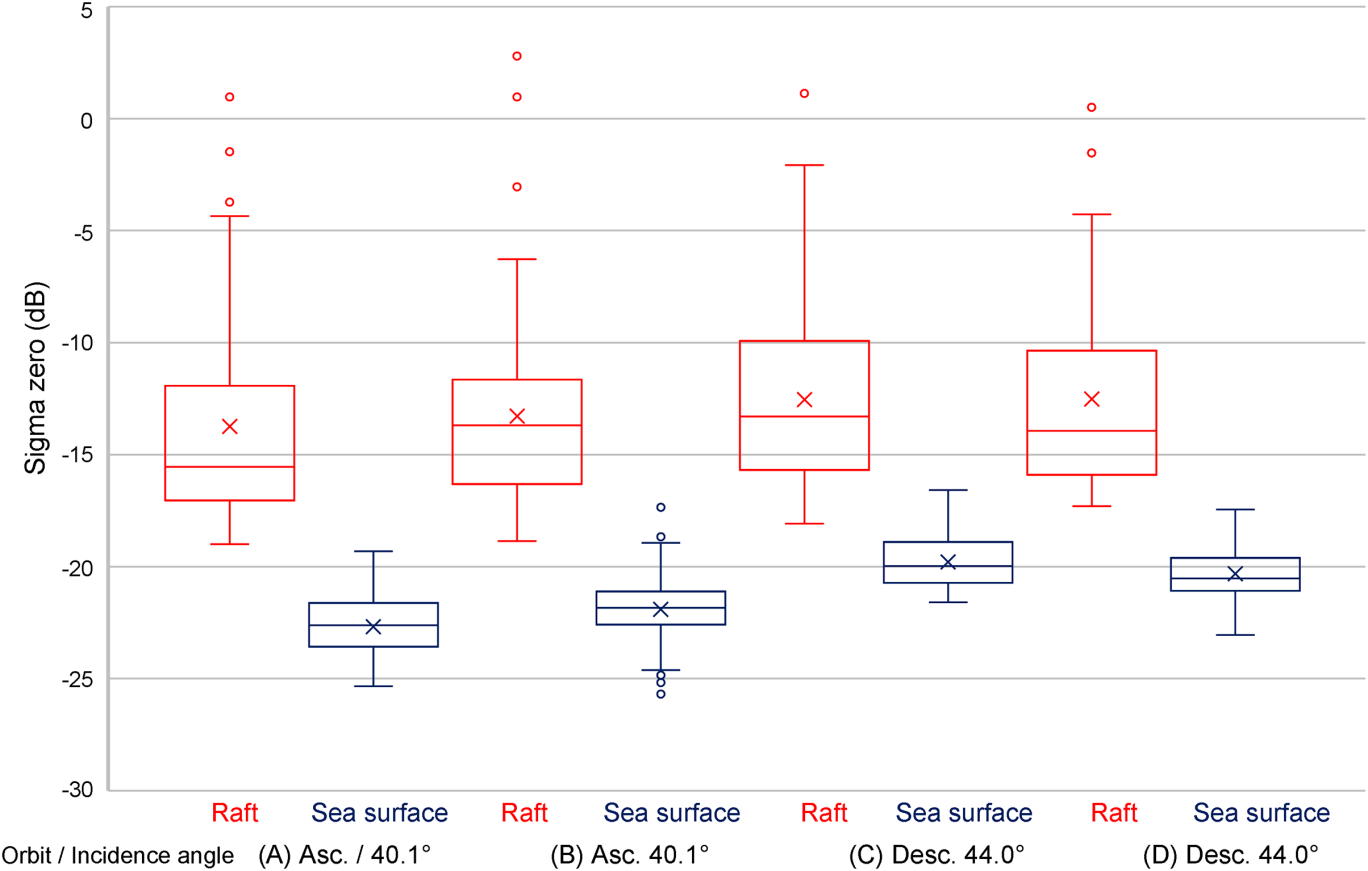
Results of sigma zero in ascending (Asc.) and descending (Desc.) orbits. Results of sigma zero which the center incidence angle was 40.1° in ascending (Asc.) orbit observation (A, B), and sigma zero the center incidence angles were 44.0° in descending (Desc.) orbit observation (C, D).

The results under the Asc. orbit with an incidence angle of 40.1°, and the results under the Desc. orbit with an incidence angle of 44.0°, are shown in Figs. 4 and 5, and Table 3. The overlap was observed between the aquaculture raft and sea surface ${\sigma ^0}$ range in the two data collected on November 24, 2021, and March 23, 2021. Result of the data from same orbit at different seasons showed median of ${\sigma ^0}$ difference in aquaculture rafts or the sea surface within 0.5–1.8 dB. However, it was difficult to assess whether these data were peculiar or not only from this result.

**Table 3:** Summary of sigma zero (dB) results under ascending (Asc.) and descending (Desc.) orbits in two different seasons data.

Orbit	Asc.				Desc.			
Date	March 3, 2021		November 24, 2021		March 23, 2021		December 28, 2021	
Incidence angle (°)	40.1				44.0			
Object	Aquaculture raft	Sea surface	Aquaculture raft	Sea surface	Aquaculture raft	Sea surface	Aquaculture raft	Sea surface
Max	1.0	−19.3	2.8	−17.4	1.1	−16.6	0.5	−17.4
Min	−19.0	−25.3	−18.8	−25.7	−18.1	−21.6	−17.3	−23.1
Median	−15.5	−22.6	−13.7	−21.8	−13.3	−20.0	−13.9	−20.5
Standard deviation	4.7	1.4	4.6	1.6	3.9	1.2	4.5	1.1

### Differences in sigma zero due to different incidence angles under the Desc. orbit

**Figure 6:**
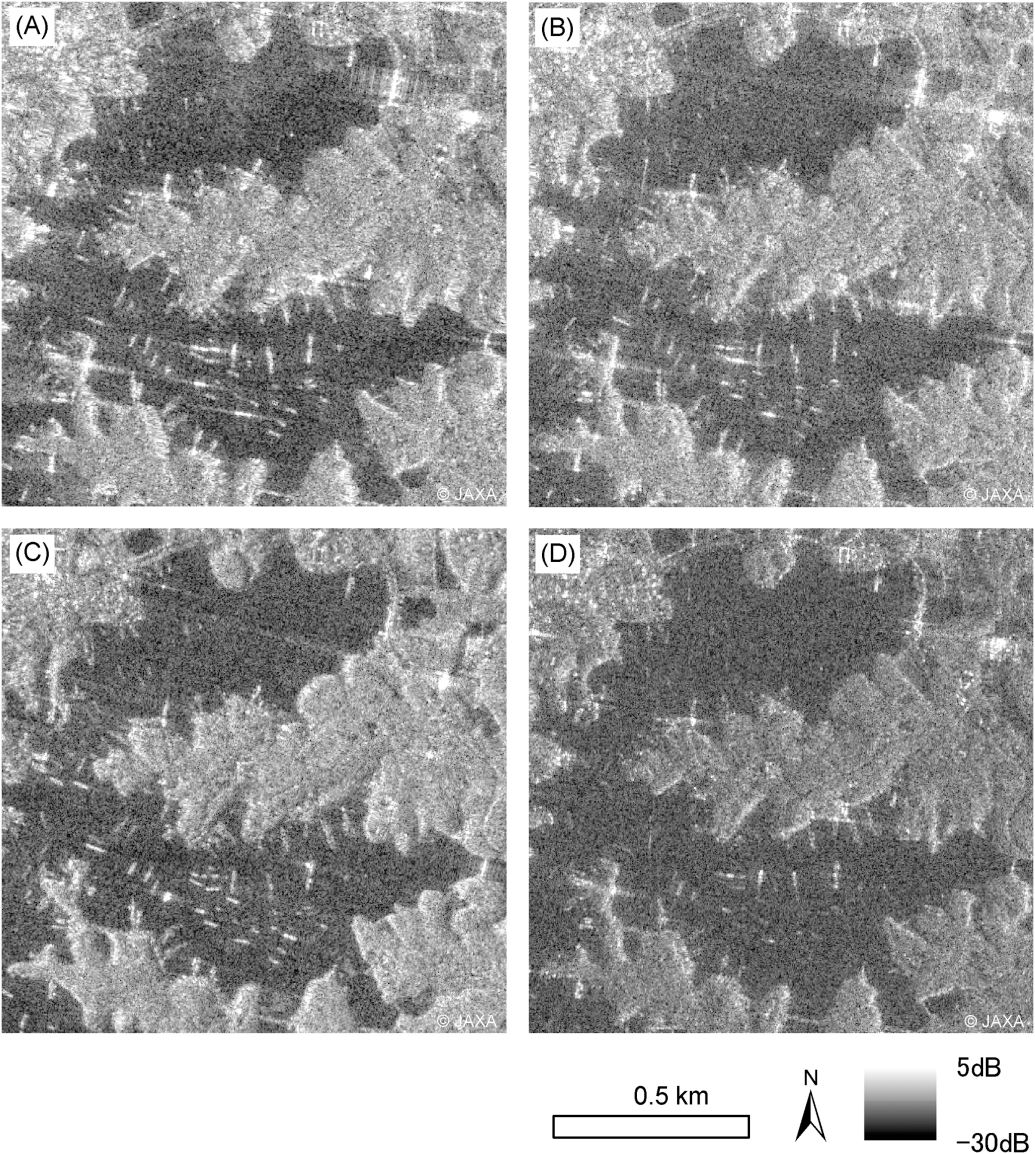
ALOS-2 PALSAR-2 HH single polarization images in descending orbit observation. The center incidence angle was 33.8° on August 1, 2021 (A), 44.0° on September 7, 2021 (B), 45.1° on May 25, 2021 (C), and 52.1° on December 9, 2021 (D). ALOS-2 PALSAR-2 data copyrights belong to Japan Aerospace Exploration Agency (JAXA).

**Figure 7:**
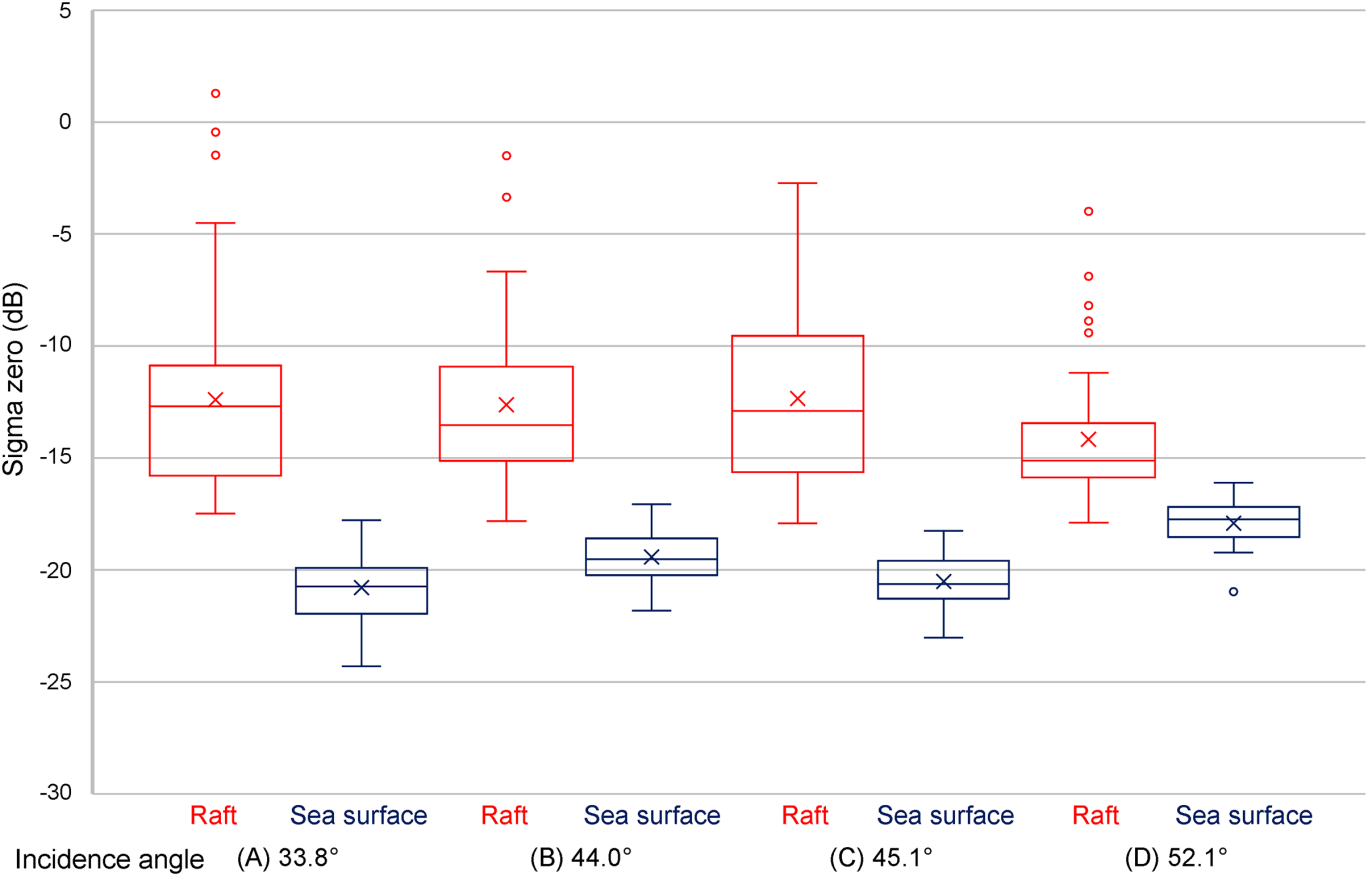
Results of sigma zero in different center incidence angles (33.8°, 44.0°, 45.1°, and 52.1°) under descending orbit observation.

The results under the Desc. orbit with different incidence angles are shown in Figs. 6, 7 and Table 4. We observed a difference in the median of ${\sigma ^0}$ between the incidence angles of the aquaculture rafts. Notably, while the incidence angle was between 33.8° to 45.1°, and the median of ${\sigma ^0}$ was between −12.7 dB to −13.5 dB, the incidence angle was 52.1°, and the median of ${\sigma ^0}$ was −15.1 dB. However, in two of the four datasets, overlap in the ${\sigma ^0}$ range between the aquaculture raft and the sea surface was observed. Still, the ${\sigma ^0}$ range was not overlapped under the incidence angles 33.8° and 45.1°, with distinct differences in the median of ${\sigma ^0}$ being observed between the aquaculture rafts and the sea surface. While the incidence angle was between 33.8° to 45.1°, and the ${\sigma ^0}$ difference was 6.0 dB to 8.1 dB, the incidence angle was 52.1°, and the ${\sigma ^0}$ difference was 2.6 dB.

**Table 4:** Summary of sigma zero (dB) results with different center incidence angles under descending (Desc.) orbit.

Orbit	Desc.							
Date	August 1, 2021		7 September, 2021		25 May, 2021		9 December, 2021	
Incidence angle (°)	33.8		44.0		45.1		52.1	
Object	Aquaculture raft	Sea surface	Aquaculture raft	Sea surface	Aquaculture raft	Sea surface	Aquaculture raft	Sea surface
Max	1.3	−17.8	−1.5	−17.1	−2.7	−18.3	−4.0	−16.1
Min	−17.5	−24.3	−17.8	−21.8	−17.9	−23.0	−17.9	−21.0
Median	−12.7	−20.7	−13.5	−19.5	−12.9	−20.6	−15.1	−17.7
Standard deviation	4.2	1.4	3.6	1.1	3.8	1.1	2.8	1.0

### Differences in sigma zero due to the direction of the aquaculture rafts

**Figure 8:**
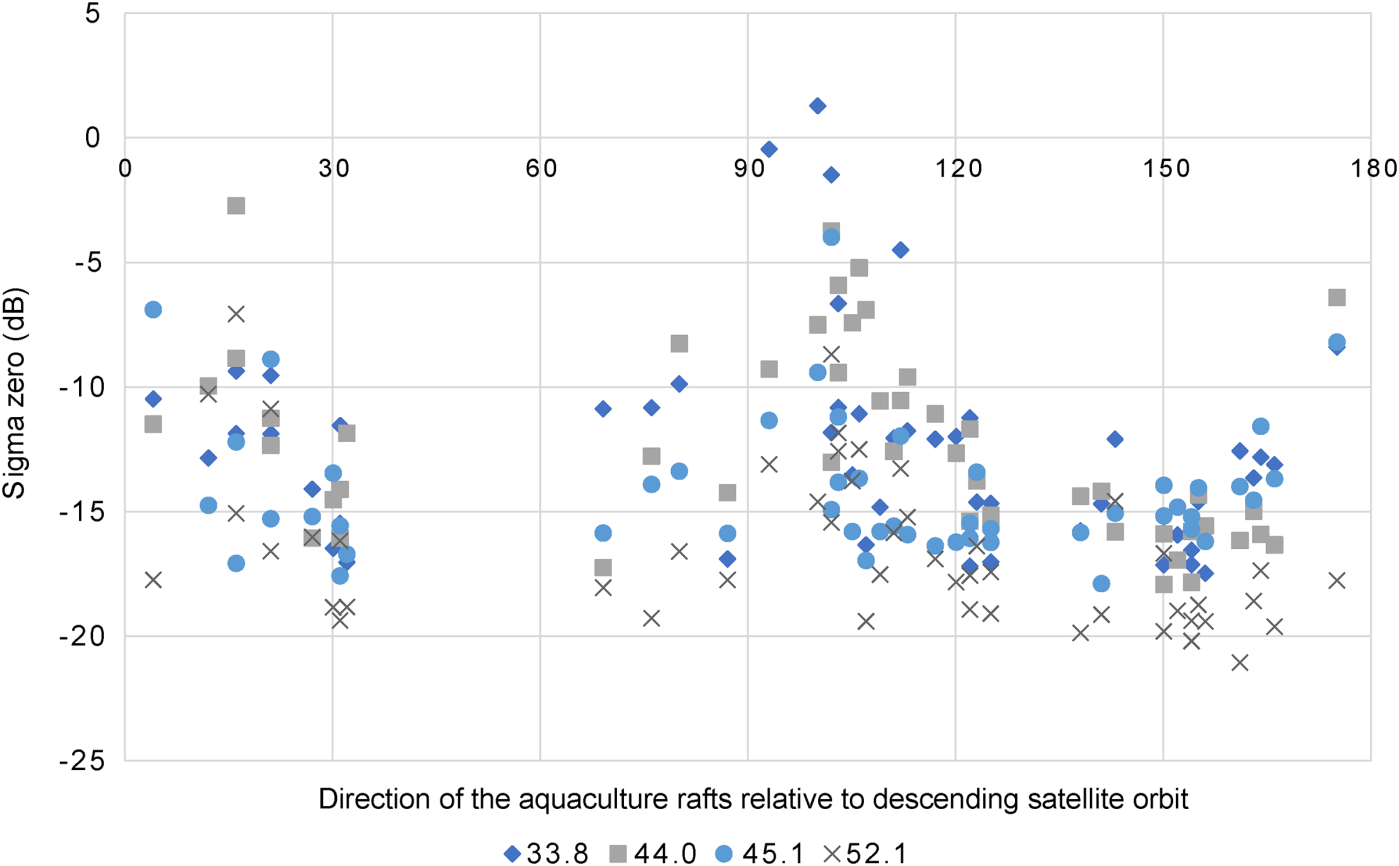
Results of sigma zero and aquaculture rafts direction. Results of sigma zero and aquaculture rafts direction to descending satellite orbit in different center incidence angles (33.8°, 44.0°, 45.1°, and 52.1°). Analyzed aquaculture rafts are same as shown in Fig. 6.

The results of ${\sigma ^0}$ due to the direction of the aquaculture rafts relative to the satellite orbit direction are plotted in Fig. 8. Notably, high values of ${\sigma ^0}$ were found when the direction was close to 90°, with the three highest ${\sigma ^0}$ of the aquaculture raft’s direction being 93.1°, 100.1°, and 102.1°, and the incidence angle being at 33.8°. Investigations also revealed that seven plotted results had ${\sigma ^0}$ higher than −5 dB, with the incidence angles being four at 33.8°, two at 44.0°, one at 45.1°, and none at 52.1°. Moreover, the two plots at incidence angles of 33.8° and 45.1° were the same aquaculture raft, whose direction was 102.1°.

## Discussion

### The effect of satellite orbit’s direction difference

Mahdavi, Amani & Maghsoudi (2019) analyzed the backscatter of a tree, ground vegetation, and water area using space-borne C-band SAR RADARSAT-2 full polarimetric images of Asc. and Desc. orbits whose incidence angles were both between 22.1° (lower edge) and 24.1° (higher edge). They reported differences in backscatter between the Asc. and Desc. orbits. The image observed in Asc. orbit was generally higher than the image acquired in the Desc. orbit, and the average difference was more than 1 dB, except for the water area.

In this study, however, the results differed from the previous study. Our investigations showed that the median of ${\sigma ^0}$ under the Desc. orbit, aquaculture rafts and sea surfaces were maximum 2.2 dB and 2.6 dB higher than those under the Asc. orbit, respectively. However, it was difficult to conclude that the difference in ${\sigma ^0}$ between the Asc. and Desc. orbits was caused only by orbital differences due to the incidence angle differed from 40.1° in the Asc. orbit and 44.0° in the Desc. orbit. Therefore, we tried to analyze data at similar incidence angles in the Asc. and Desc. orbits to clarify this gap. Still, there were no data on them on this study site.

### The effect of aquaculture raft direction, satellite orbit direction, and Bragg resonance scattering appearance on aquaculture rafts

**Figure 9:**
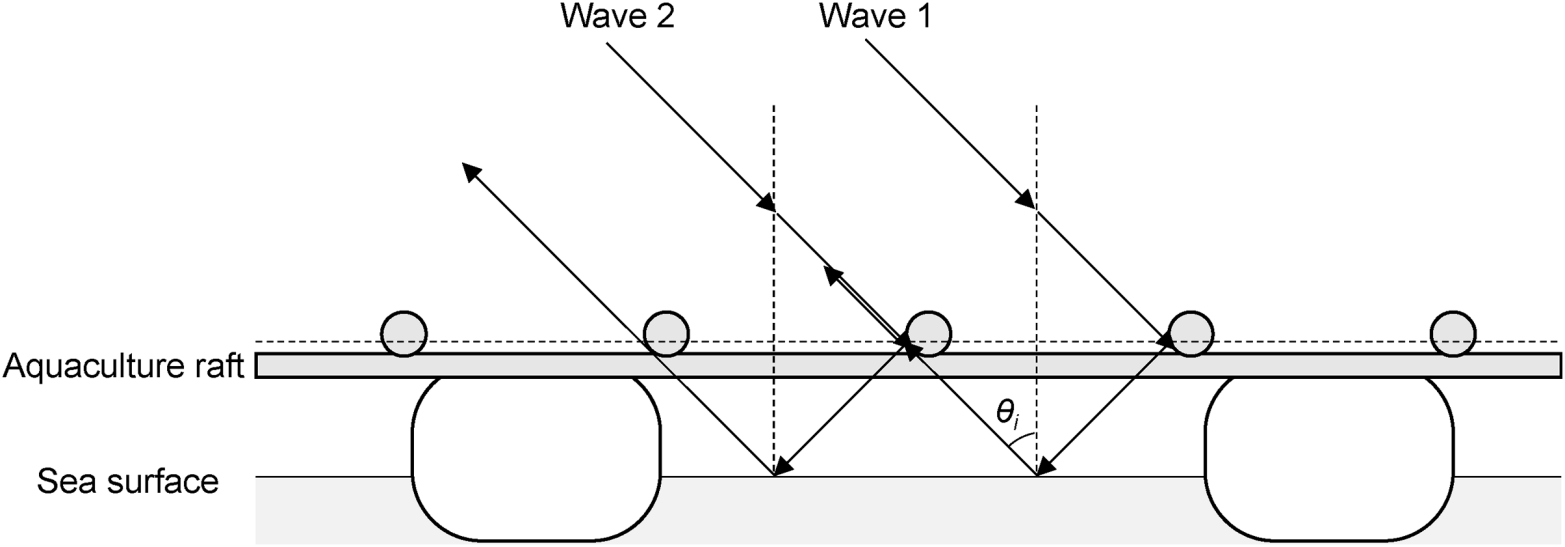
Scattering paths of two incident waves on aquaculture raft.

Since the ${\sigma ^0}$ from aquaculture rafts are caused by a reflection from the wood poles or buoys that are to construct these aquaculture rafts and the sea surface inside them, differences in the ${\sigma ^0}$ of aquaculture rafts may be attributed to these differences. The outstandingly high value of ${\sigma ^0}$ for several aquaculture rafts compared to the median of ${\sigma ^0}$ were found when the aquaculture raft direction was close to the perpendicular (90°) of the satellite orbit direction, and those incidence angle was 33.8°, 44.0°, and 45.1°. We consider this mechanism as Bragg scattering. In previous studies, Rosenqvist (1999) and Ouchi, Niiuchi & Mohri (1999) reported Bragg scattering from rice plants using JERS-1 L-band SAR images of mechanically planted rice fields. Their results showed strong radar backscattering if the planting orientation, incidence angle, and distance between the bunches of rice plants matched the Bragg resonance condition. The Bragg resonance condition was defined by Rosenqvist (1999), as shown in Eq. (2). Ouchi et al. (2006) later reported the mechanism of Bragg resonance scattering, which arises from the double-bounce scattering between water surfaces and the regularly spaced bunches of rice plants.

(2) $${d_{max}} = n\gamma {\left( {2sin{\theta _i}} \right)^{ - 1}}$$

where *d* is the plant spacing, $\gamma$ is the radar wavelength, and ${\theta _i}$ is the local incidence angle. In this equation, *d* could replace the distance of wood poles that make up the aquaculture raft, and $\gamma$ = 22.9 cm for ALOS-2 PALSAR-2. Furthermore, the incidence angle was 33.8°, 40.1°, 44.0°, 45.1°, and 52.1°. For example, when *n* = 3 in Eq. (2), *d* = 0.62 m, 0.53 m, 0.49 m, 0.48 m, and 0.44 m, respectively. In this case, the incidence angle of 33.8° was close to 0.64 m, the distance of wood poles. Since the aquaculture rafts were made by hand, there were slight differences in the distance of wood poles. In addition, it has an effect from the sea surface. Based on this finding, we propose that the roughness of the sea surface changed the reflection and effect to appearance of the Bragg resonance scattering on the aquaculture raft. In this study, the ${\sigma ^0}$ of the aquaculture rafts were considered includes the effect of Bragg resonance scattering that means double-bounce scattering occurred between the aquaculture raft and the sea surface, resulting in a high value of ${\sigma ^0}$ (Fig. 9). Therefore, Bragg resonance scattering was considered to make detecting the aquaculture rafts from the sea surface easier. Moreover, if the distance between wood poles is known, the theoretical incidence angle can be calculated to obtain the Bragg resonance scattering.

### The effect of incidence angles on detecting aquaculture rafts

Skrunes et al. (2018) studied the backscatter of clean sea surfaces at different incidence angles using UAV-based SAR. They reported that the clean sea backscatter decreased as the incidence angle increased in HH polarization. Studies on sea ice using SAR have also reported the incidence angle dependence of backscatter. For example, Mahmud et al. (2018) used L-band SAR ALOS PALSAR and RADARSAT-2 and investigated the incidence angle dependence of backscatter over Arctic sea ice. They analyzed Arctic first-year ice and multiyear sea ice and reported mean ice type-specific incidence angle dependencies were −0.21 dB/1° and −0.30 dB/1°, respectively for ALOS, and −0.22 dB/1° and −0.16 dB/1°, respectively for RADARSAT-2. They concluded that it must normalize SAR images calculated by incidence angle dependence to improve sea ice classification. In this study, the aim is to detect aquaculture rafts daily and immediately after the disaster. Therefore, incidence angle normalization is not necessary, but it is necessary to recognize the ${\sigma ^0}$ value depends on the incidence angle and the effects of the ground surface. Singha, Johansson & Doulgeris (2020) used L-band SAR ALOS-2 PALSAR-2 full polarimetric images with a range of incidence angles and during different environmental conditions for artificial neural network-based sea ice type classification. The highest incidence angle (39.05°) they used has values deviating from the otherwise consistent trend, including previous studies (Casey et al., 2016; Wakabayashi et al., 2004; Mahmud et al., 2018) with decreasing backscattering value with the incidence angle. This was considered since rough surfaces were more prominent at higher incidence angles due to different imaging geometries and different proportions of ice types in those scenes.

Alternatively, this study showed that although the median of ${\sigma ^0}$ on the sea surface was almost the same (−20.7 dB and −20.6 dB), even when the incidence angles were 33.8° and 45.1° (11.3° difference), respectively, ${\sigma ^0}$ did not decrease with an increasing incidence angle, as reported in previous studies. Furthermore, the median of ${\sigma ^0}$ on the sea surface was −17.7 dB when the incidence angle was 52.1°, higher than the incidence angle of 33.8° and 45.1°. Therefore, we propose that this finding was due to the roughness of the sea surface, confirming the results of Singha, Johansson & Doulgeris (2020), which indicated that when the sea surface is rough, ${\sigma ^0}$ indicates high value prominence at higher incidence angles, making it difficult to distinguish them from aquaculture rafts.

We observed that when the incidence angle was 44.0° on September 7, 2021, the ${\sigma ^0}$ range of the aquaculture raft and sea surface was overlapped. However, when the incidence angle was 44.0° on December 28, 2021, the ${\sigma ^0}$ range did not overlap. The incidence angle of 44.0° on September 7, 2021, was attributed to the roughness of sea surfaces, such as rain, wave, and other movements. Furthermore, the median of ${\sigma ^0}$ on aquaculture raft at an incidence angle 52.1° was obviously lower than that at 33.8°–45.1°. Therefore, an incidence angle between 33.8° and 45.1° was considered optimum for detecting aquaculture rafts with high ${\sigma ^0}$ values, minimizing the effects of sea surface roughness. However, since there are no data on incidence angles <33.8°, further data and analysis are still required.

## Conclusions

This study analyzed ALOS-2 PALSAR-2 data to examine (1) differences in ${\sigma ^0}$ between the Asc. and Desc. orbits of the satellite, (2) differences in ${\sigma ^0}$ due to different incidence angles under the Desc. orbit, and (3) differences between the aquaculture raft and satellite orbit directions in Ago Bay, Mie Prefecture, central Japan, to evaluate the effect of the imagery’s incidence angle on the ability to detect aquaculture rafts. During emergency observations, although selecting an orbit is impossible immediately after a disaster, it is possible to customize the incidence angle. Nevertheless, the aquaculture rafts size smaller than the spatial resolution of SAR data may become difficult to detect, and if the sea surface is rough, it also become difficult to detect aquaculture rafts. When the aquaculture raft’s direction is perpendicular to the satellite orbit, Bragg resonance scattering is more likely to occur, making detection easier. In addition, suppose the precise size of the aquaculture raft is known; the theoretical incidence angle can also be calculated to cause Bragg resonance scattering, which can then be applied to enhance the ability to detect individual aquaculture rafts.

Monitoring aquaculture rafts using SAR data is crucial for stable-farmed seafood supply because it is an all-weather sensor; several countries now plan L-band SAR missions. For instance, while the European Space Agency has prepared ROSE-L (Radar Observing System for Europe-L-band) or Sentinel 12 in the 2020s, the National Aeronautics and Space Administration and Indian Space Research Organization SAR (NISAR) introduced a joint mission NISAR, employing L-band SAR for their investigations. These L-band SAR data applying for coastal management can expect. For future studies, continuous observation with several incidence angles between 33.8° and 45.1° is considered appropriate for these SAR sensors.

## Additional Information and Declarations

### Data Availability

The following information was supplied regarding data availability: The ALOS-2 PALSAR-2 data is available at the Earth Observation Data Utilization Promotion Platform (https://satpf.jp/spf/?lang=en) and G-Portal (https://gportal.jaxa.jp/gpr/?lang=en) which requires registration. Image ID: ALOS2366020670-210303 (March 3, 2021). Image ID: ALOS2368902930-210323 (March 23, 2021). Image ID: ALOS2378222880-210525 (May 25, 2021). Image ID: ALOS2388272920-210801 (August 1, 2021). Image ID: ALOS2393742930-210907 (September 7, 2021). Image ID: ALOS2405350670-211124 (November 24, 2021). Image ID: ALOS2407492930-211209 (December 9, 2021). Image ID: ALOS2410302930-211228 (December 28, 2021).
